# ALDH1A3: A Marker of Mesenchymal Phenotype in Gliomas Associated with Cell Invasion

**DOI:** 10.1371/journal.pone.0142856

**Published:** 2015-11-17

**Authors:** Wenlong Zhang, Yanwei Liu, Huimin Hu, Hua Huang, Zhaoshi Bao, Pei Yang, Yinyan Wang, Gan You, Wei Yan, Tao Jiang, Jiangfei Wang, Wei Zhang

**Affiliations:** 1 Department of Neurosurgery, Beijing Tiantan Hospital, Capital Medical University, Beijing, China; 2 Department of Molecular Neuropathology, Beijing Neurosurgical Institute, Capital Medical University, Beijing, China; 3 Department of Neurosurgery, the First Affiliated Hospital of Nanjing Medical University, Nanjing, China; Cedars-Sinai Medical Center, UNITED STATES

## Abstract

Aldehyde dehydrogenases (ALDH) is a family of enzymes including 19 members. For now, ALDH activity had been wildly used as a marker of cancer stem cells (CSCs). But biological functions of relevant isoforms and their clinical applications are still controversial. Here, we investigate the clinical significance and potential function of ALDH1A3 in gliomas. By whole-genome transcriptome microarray and mRNA sequencing analysis, we compared the expression of ALDH1A3 in high- and low- grade gliomas as well as different molecular subtypes. Microarray analysis was performed to identify the correlated genes of ALDH1A3. We further used Gene Ontology (GO) and Kyoto Encyclopedia of Genes and Genomes (KEGG) pathways analysis to explore the biological function of ALDH1A3. Finally, by mRNA knockdown we revealed the relationship between ALDH1A3 and the ability of tumor invasion. ALDH1A3 overexpression was significantly associated with high grade as well as the higher mortality of gliomas in survival analysis. ALDH1A3 was characteristically highly expressed in Mesenchymal (Mes) subtype gliomas. Moreover, we found that ALDH1A3 was most relevant to extracellular matrix organization and cell adhesion biological process, and the ability of tumor invasion was suppressed after ALDH1A3 knockdown in vitro. In conclusion, ALDH1A3 can serve as a novel marker of Mes phenotype in gliomas with potential clinical prognostic value. The expression of ALDH1A3 is associated with tumor cell invasion.

## Introduction

ALDH is a family of enzymes including 19 members that metabolize both endogenous and exogenous aldehydes to carboxylic acids as well as other reactive compounds. It is becoming increasingly evident that ALDH activity, originally used for the isolation of hematopoietic stem cells, is a hallmark of cancer stem cells (CSC) measurable by the aldefluor assay [1]. Furthermore, high activity of ALDH was associated with poor prognosis in breast cancer, bladder cancer, and prostate cancer patients [2–4]. Though generally believed to be responsible for the ALDH activity of CSCs of many cancers, ALDH1A1 was a poor prognostic indicator in gliomas [5, 6]. More recently, several studies indicated that other ALDH isoforms, particularly ALDH1A3, significantly contributed to aldefluor positivity. Moreover, ALDH1A3, as a novel marker of a CSC, could predict metastasis and/or clinical prognosis in many cancers [7–9]. In our previous study, we have found that hypermethylation status of ALDH1A3 promoter predicted a better prognosis with an accompanied low expression of ALDH1A3 protein in G-CIMP-negative primary GBMs [10].

Based on mRNA expression microarray, The Cancer Genome Atlas (TCGA) has divided GBM into four subtypes: Proneural (PN), Neural, Classical, and Mesenchymal (Mes), which are widely accepted nowadays[11]. One recent study revealed that glioma stem cells (GSC) could also be classified into two distinct subtypes (PN GSCs and Mes GSCs). ALDH1A3 was defined as a biomarker of Mes GSCs. ALDH1A3 knockdown would inhibit PN-to-Mes transformation which is induced by radiation treatment [12]. In addition to be a novel marker of GSCs, ALDH1A3 is likely to play an important role in glioma, especially in a subtype dependent manner.

In this study, by whole-genome transcriptome microarray and mRNA sequencing analysis, we compared the mRNA expression level of ALDH1A3 in high- and low- grade gliomas and different molecular subtypes. The relationship between ALDH1A3 mRNA expression and clinical outcome was also evaluated. Finally, functional experiments in vitro revealed that ALDH1A3 could not only be a novel biomarker of Mes phenotype in gliomas, but was correlated with tumor cell invasion.

## Materials and Methods

### Patients and samples

Six hundred and twenty-six tumor specimens were included in our study. All the glioma patients underwent surgical resection and subsequently received radiation therapy and/or alkylating agent based chemotherapy. The clinical characteristics of patients were listed in Table 1. According to the 2007 WHO classification guidelines [13], the diagnosed gliomas were re-reviewed in histological slides by two experiential neuropathologists. This study was approved by the Ethics Committee of Beijing Tiantan Hospital and written informed consent was obtained from all patients.

**Table 1:** Clinical characteristics of patients.

	Whole-genome expression profiling (n = 301)	Whole transcriptome sequencing (n = 325)
Age-year		
Median	43	43
Age—no. (%)		
<40 year	131(43.5)	125 (38.5)
40–60 year	139 (46.2)	164 (50.5)
≥60 year	31(10.3)	36 (11.1)
Sex-no. (%)		
Male	180 (58.4)	203 (62.5)
Female	121 (41.6)	122 (37.5)
WHO grade-no. (%)		
II	122 (40.5)	109 (33.5)
III	51 (16.9)	72 (22.2)
IV	128 (42.5)	144 (44.3)
TCGA subtypes		
Proneural	89 (29.6)	102 (31.4)
Neural	61 (20.3)	81 (24.9)
Classical	46 (15.3)	74 (22.8)
Mesenchymal	105 (34.9)	68 (20.9)

### Whole transcriptome sequencing and whole-genome expression profiling

325 tumor samples (109 with grade II, 72 with grade III and 144 with grade IV) were performed whole transcriptome sequencing, and 301 samples (122 with grade II, 51 with grade III and 128 with grade IV) were performed whole-genome expression profiling. The detailed procedures had been described in our previously published works [14–16]. Besides, the data we generated can be retrieved partly on the public platforms Chinese Glioma Genome Atlas (CGGA) (http://www.cgga.org.cn). 441 GBM expression files from the cancer genome atlas (TCGA) database (http://cancergenome.nih.gov).

In CGGA genome-wide transcriptome microarray data, microarray analysis was performed on all samples using the Agilent Whole Human Genome Array according to the manufacturer’s instructions. The integrity of total RNA was checked using an Agilent 2100 Bioanalyzer (Agilent). cDNA and biotinylated cRNA were synthesized and hybridized to the array. Data were acquired using the Agilent G2565BA Microarray Scanner System and Agilent Feature Extraction Software (version 9.1). Probe intensities were normalized using GeneSpring GX 11.0 [14]. Illumina HiSeq2000 sequencing system was used to acquire whole transcriptome sequencing data, which were subsequently mapped to the human reference gene set and reference genome RefSeq (hg19) using BWA[16]. Reads Per Kilobase per Million (RPKM) was calculated as a measure of relative abundance of transcripts[17]. All gene expression data presented above in this paper was normalized and log transformed.

For TCGA data, HT-HG-U133A gene expression array platform was used and the data were preprocessed using quantile normalization and RMA through the aroma package, in combination with a gene-centric CDF[11].

### Cell lines and transfection

The human glioma cell lines U87 and LN229, obtained from the Institute of Biochemistry and Cell Biology, Chinese Academy of Science, were used. They were maintained in Dulbecco’s modified Eagle’s medium (DMEM, Hyclone) containing 10% heat-inactivated fetal bovine serum (FBS, Hyclone), 50 U/ml penicillin G, and 250 μg/ml streptomycin in a humidified atmosphere containing 5% CO_2_ at 37°C. Three groups were set up in this study, including ALDH1A3 shRNA group, negative control group and wild type group. For shRNA transfection, the designed shRNAs were synthesized by GenePharma (Shanghai, China). The targeted sequence of ALDH1A3 was sense 5’-GCAGGTCTACTCTGAGTTTGT -3’. An ineffective shRNA cassette in the Vector (GenePharma) was used as a negative control. The wild type group means treating the cell lines with Lipofectamine 2000 only. Transfection was performed using Lipofectamine 2000 Transfection Reagent (Invitrogen, China) according to the manufacturer’s instructions. The cells were collected and subjected to subsequent analysis 48 h after transfection.

### Western blotting

The cells were collected and lysed in RIPA buffer (Cell Signaling Technology, USA). Homogenates of cells were clarified by centrifugation at 14,000 x g for 15 min at 4°C. The supernatants were collected, and the protein concentrations were measured by the BCA Protein Assay Kit (Sigma, USA). SDS-PAGE was performed on 40 μg of protein from each sample, gels were transferred to PVDF membranes (Millipore, USA) and incubated with the anti-GAPDH (Sigma, USA), anti-ALDH1A3 (Abcam, UK), anti-MMP2 (Abcam, UK), anti-snail and anti-slug (Cell Signaling Technology, USA). Followed by incubation with Peroxidase-Conjugated AffiniPure secondary antibody (ZSGB-BIO, China), the protein bands in the membranes were developed using Bio-Rad GelDoc XR System and analyzed by Image Lab 5.0 (Bio-Rad laboratories).

### Transwells

Transwell plates (24-well, 8-μm pore size, Corning, USA) with a Matrigel (BD Bioscience, USA) coating layer were used to assess the cell invasive and migratory abilities. Briefly, U87 and LN229 cells were pretreated with ALDH1A3 shRNA or negative control or none for 48 h. Then they were seeded and plated at 5 × 10^4^ cells/well in the upper transwell chambers of inserts precoated with matrigel in serum and growth-factor-free medium. The plates were incubated at 37°C for 24 h and 36 h in with 5% CO_2_ and then stained with crystal violet. Invading cells were defined as cells that had degraded the Matrigel and moved into the lower surface of the membrane, and the non-invading cells which were retained on the upper surface of the membrane were removed by a cotton swab. Cells were counted in triplicate membranes.

### Statistical analysis

Genes harboring positive and negative correlation with ALDH1A3 mRNA expression were calculated using Matlab software. GO and KEGG pathways analysis was performed using DAVID (http://david.abcc.ncifcrf.gov). Kaplan-Meier survival analysis was used to estimate the survival distributions. Patients with overall survival equal or greater than 60 days were involved. The log-rank test was used to assess the statistical significance between stratified survival groups with use of GraphPad Prism, version 5.0 statistical software. Student’s two-tailed *t* test was used to determine significant differences. All data are presented as the mean ± standard error. All values were considered statistically significant at p<0.05. Prediction Analysis of Microarrays was used to annotate the CGGA samples with PN, Neural, Classical, and Mes labels[18].

## Results

### ALDH1A3 is associated with the malignant phenotype in gliomas

**Fig 1:**
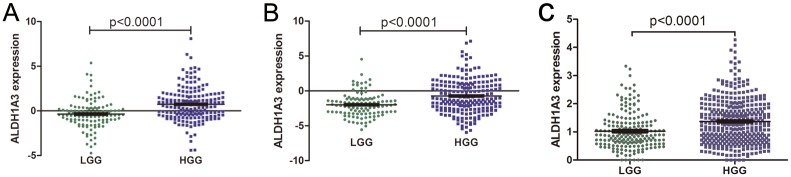
Correlation of ALDH1A3 mRNA expression with tumor malignancy. **A**. By genome-wide transcriptome microarray analysis, ALDH1A3 expressed higher in HGG (n = 179) than in LGG (n = 122) (p<0.0001). **B**. By whole transcriptome sequencing analysis, ALDH1A3 mRNA was overexpressed in HGG (n = 216) than in LGG (n = 109) (p<0.0001). Lines in the middle were the mean expression value. **C**. In TCGA whole transcriptome sequencing date, ALDH1A3 mRNA was overexpressed in HGG (n = 193) than in LGG (n = 374) (p<0.0001).

In previous studies, ALDH1A3 and ALDH1A1 were supposed to be two important ALDH isoforms in gliomas [5, 6, 10, 12]. We speculated that they might be associate with the malignancy of glioma. By whole-genome expression profiling data, we compared the mRNA expression level of ALDH1A3 and ALDH1A1 in low-grade gliomas (LGG, n = 122) and high-grade gliomas (HGG, n = 179). The results showed that ALDH1A3 mRNA expression level was significantly up-regulated in the HGG compared with LGG (p<0.0001; Fig 1A). While, ALDH1A1 mRNA was expressed lower in HGG than LGG (p<0.0001; S1 Fig).

We further measured the expression levels of the two by whole transcriptome sequencing data. The same as the results above, ALDH1A3 mRNA was overexpressed in HGG (n = 216) than in LGG (n = 109) (p<0.0001; Fig 1B). The expression of ALDH1A1 was lower in HGG than in LGG (p<0.0001; S1 Fig).

Meanwhile, the mRNA expression level of ALDH1A1 and ALDH1A3 in high and low grade gliomas were compared by TCGA whole transcriptome sequencing data. 567 patients (193 with grade II, 207 with grade III and 167 with grade IV) were involved. As is shown in Fig 1C, ALDH1A3 mRNA was overexpressed in HGG than in LGG (p<0.0001). The expression of ALDH1A1 was lower in HGG than in LGG (p<0.0001; S1 Fig).

### High ALDH1A3 mRNA expression was related to poor clinical outcomes in gliomas

**Fig 2:**
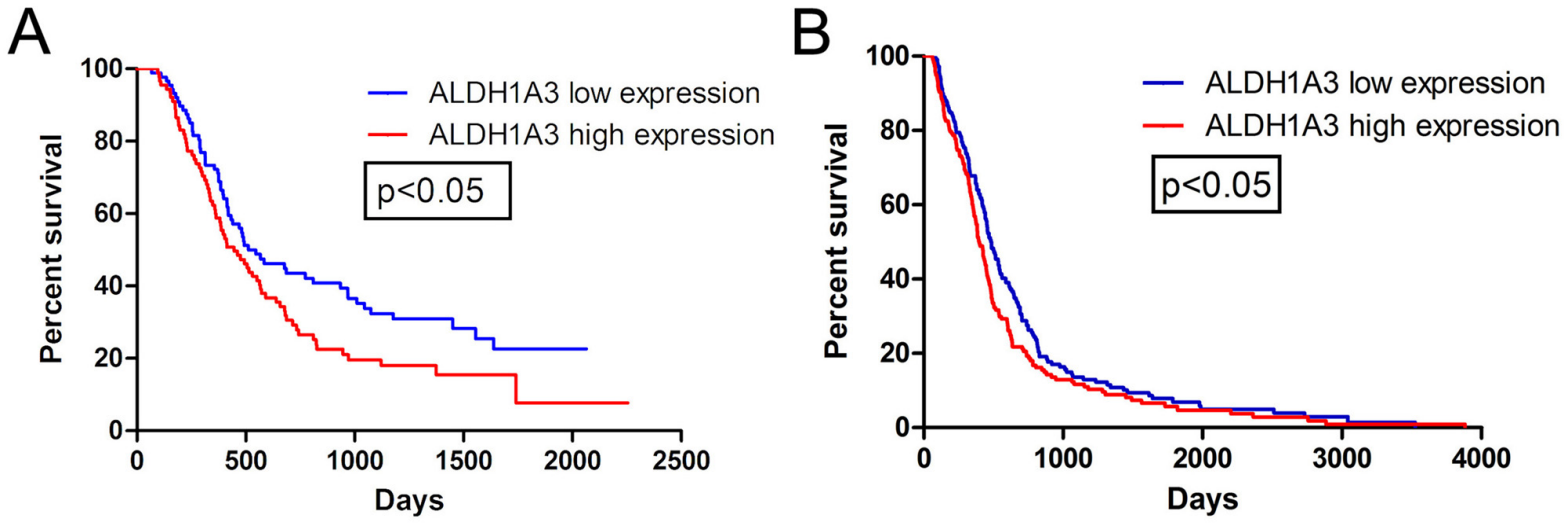
ALDH1A3 mRNA expression was related to clinical outcomes in gliomas. **A**. Kaplan-Meier estimates of survival for 177 HGG patients (OS ≥ 60 days) with genome-wide transcriptome microarray. There is a significant difference in survival between ALDH1A3 high-expression (n = 89) and low-expression patients (n = 88, p<0.05). **B**. In TCGA database, patients with ALDH1A3 high-expression (n = 220) have shorter OS than those with ALDH1A3 low-expression (n = 221, p<0.05).

The prognostic values of ALDH1A3 and ALDH1A1 were evaluated in HGG patients. From genome-wide transcriptome microarray data, 177 patients (overall survival, OS ≥ 60 days) were involved. 88 and 89 patients were classified into ALDHs low-expression group and high-expression group, respectively. Kaplan—Meier curves for ALDH1A3 exhibited significant association with OS in HGG (512 vs 447 days, p<0.05, Log-rank test; Fig 2A). However, there was no difference between ALDH1A1 high and low groups (470 vs 506 days, p = 0.5, Log-rank test; S2 Fig). We further validated the prognostic value of ALDH1A3 and ALDH1A1 by TCGA microarray database. 441 GBM patients (221 low-ALDHs vs 220 high-ALDHs, OS≥ 60 days) were included. It revealed that the mRNA expression of ALDH1A3 other than ALDH1A1 was related to patient survival (Fig 2B and S2 Fig). In conclusion, ALDH1A3 might be a novel prognostic marker in gliomas.

### ALDH1A3 can serve as a marker of Mes subtype gliomas

**Fig 3:**
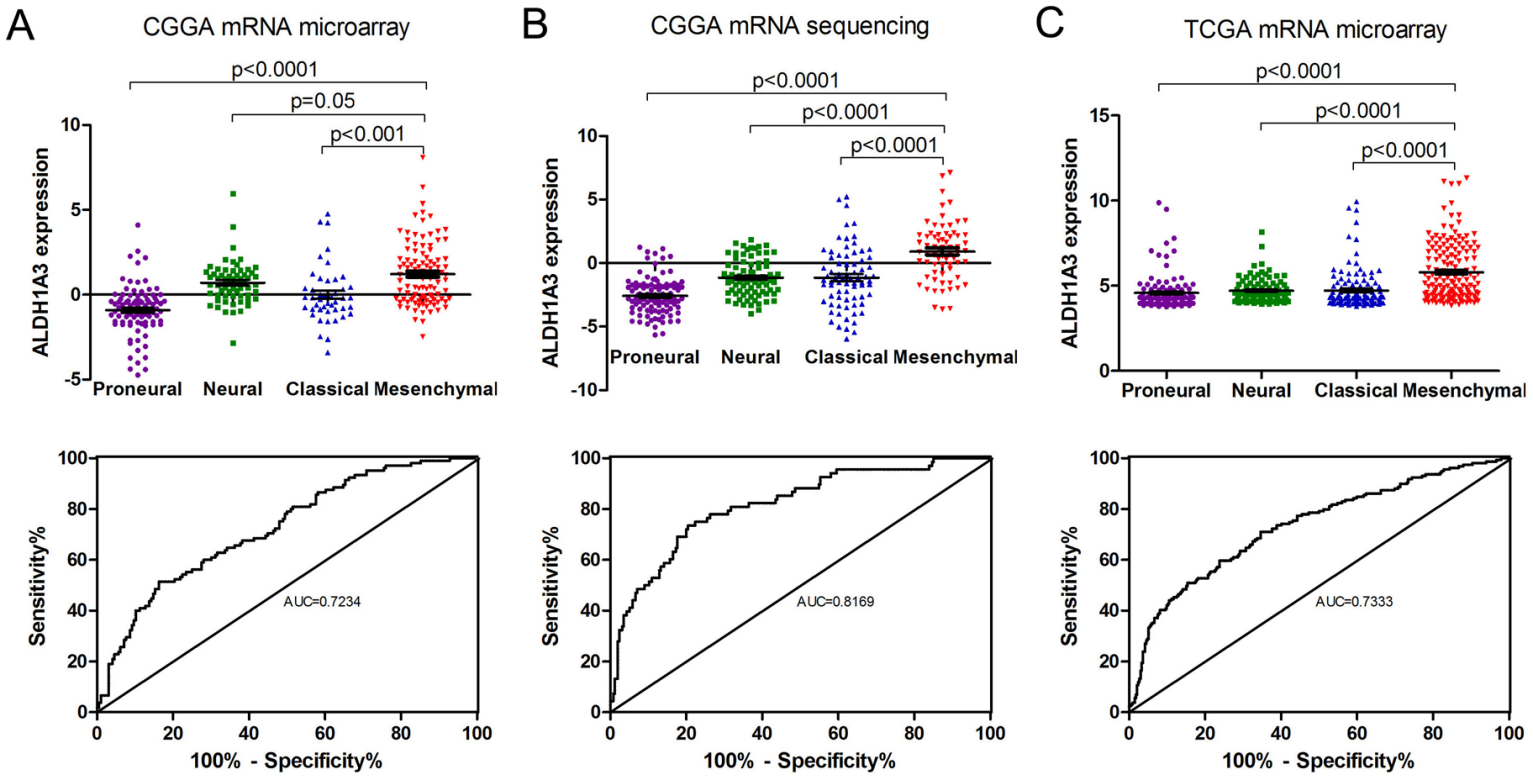
The expression of ALDH1A3 is highest in Mes subtype and can serve as a novel biomarker. **A-C**. ***upper*** The relative expression level of ALDH1A3 is evaluated in 3 databases (CGGA mRNA microarray, CGGA mRNA sequencing and TCGA mRNA microarray). The mRNA expression of ALDH1A3 is highest in Mes. Lines in the middle were the mean expression value. **A-C**. **lower** To evaluate the performance of ALDH1A3 as a biomarker of Mes subtype, ROC curve analysis was done. The score of Area Under the ROC Convex Hull (AUC) range from 0.723 to 0.817 in the 3 database.

We used our genome-wide transcriptome microarray data and sequencing data as well as TCGA microarray database to analyze ALDH1A3 mRNA expression in different molecular subtypes. As is shown in figures (Fig 3A–3C *upper*), ALDH1A3 mRNA was expressed higher in Mes subtype of gliomas than in other subtypes. For further evaluation, all the patients were divided into two groups, including Mes subtype glioma group and no-Mes subtype group. By receiver operating characteristic (ROC) analysis, we found that ALDH1A3 had a high efficiency to predict Mes subtype (Fig 3A–3C *lower*). Thus, we proposed that ALDH1A3 can serve as a novel biomarker of Mes subtype gliomas.

### ALDH1A3 is associated with cell adhesion and tumor invasion

**Fig 4:**
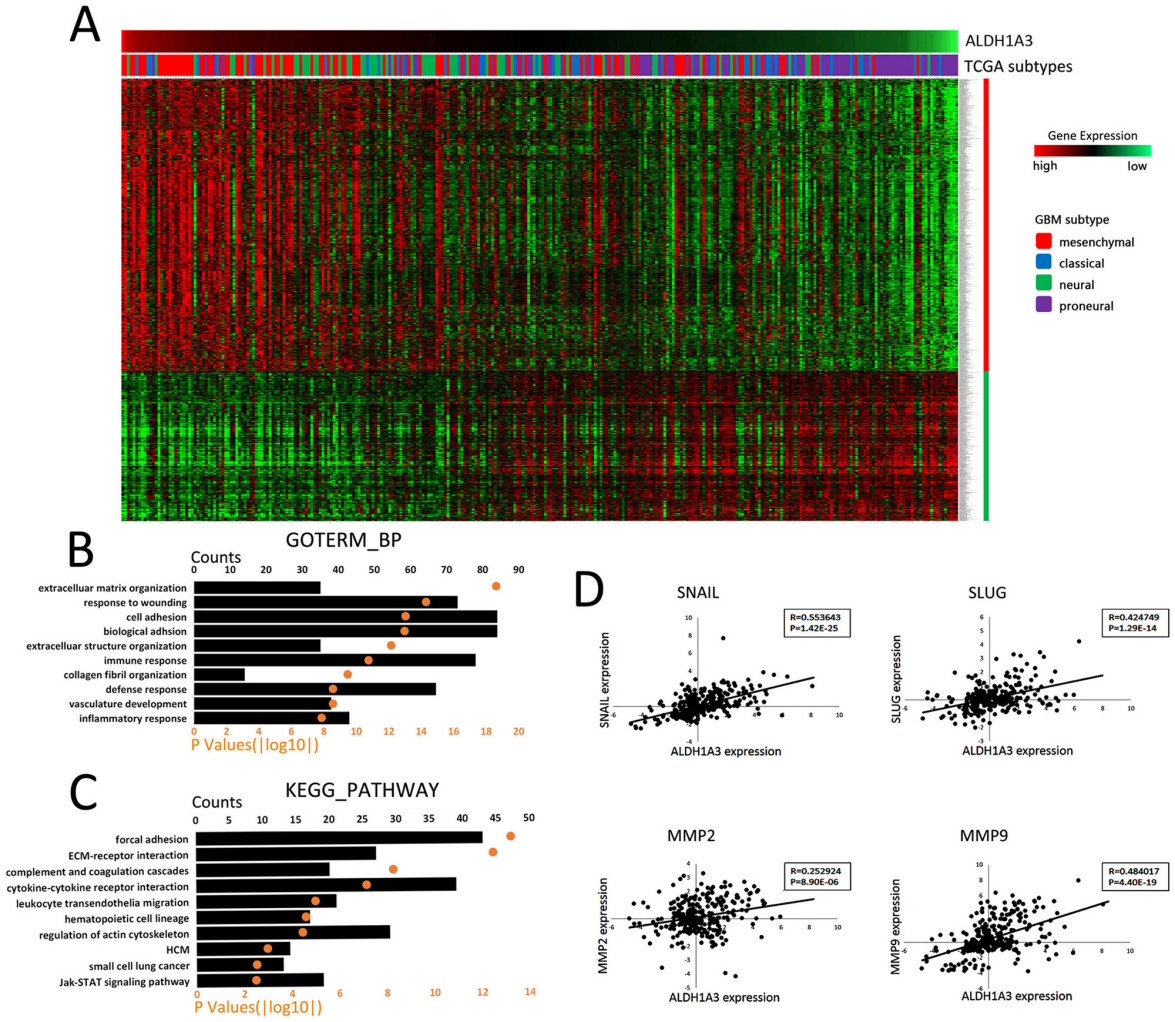
ALDH1A3 associated genes and their potential functions. **A**. 301 patients are arranged according to descending order of ALDH1A3 mRNA expression. Mes sutype patients are mainly distributed in the side of ALDH1A3 high expression. Most of the patients with ALDH1A3 relatively low expression are PN suptype. By pearson correlation analysis, 869 and 472 unique genes (1125 and 579 probes) having positive or negative relationship with ALDH1A3 mRNA expression were obtained respectively. **B, C**. GO and KEGG pathway analysis of the positively correlated genes of ALDH1A3. The upper X-axis indicates gene counts (column chart); the lower X-axis indicates p-values (scatter diagram). The top 10 potential functions and pathways have been listed in the Y-axis. **D**. ALDH1A3 has positive correlation with tumor invasion related genes, including snail, slug, MMP2 and MMP9.

As mentioned above, most of the Mes subtype patients had high ALDH1A3 mRNA expression and PN subtype patients had low ALDH1A3 mRNA expression level (Fig 4A). To further explore the potential correlated genes of ALDH1A3 in gliomas, Pearson correlation analysis was applied into CGGA whole-genome mRNA expression microarray data. There were 869 and 472 unique genes (1125 and 579 probes) with positive or negative correlation separately with ALDH1A3 mRNA expression (|R|>0.4; Fig 4A).

Gene Ontology analysis of positively correlated genes was annotated using DAVID. Top 3 GO biological processes were extracellular matrix organization (p = 2.4E-19), cell adhesion (p = 9.7E-14) and biological adhesion (p = 1.0E-13) (Fig 4B). Pathway enrichment analysis (KEGG pathways) showed that focal adhesion (p = 5.9E-14) and ECM-receptor interaction (p = 3.4E-13) were the top two associated pathways (Fig 4C). All these biological processes and signal pathway were widely thought to play important roles in tumor invasion.

Some transcription factors (such as SNAIL and SLUG) and matrix metalloproteinases (MMP2 and MMP9), were widely interpreted as the critical molecules in tumor invasion [19–22]. By Pearson correlation analysis in our mRNA expression microarray, mRNA expression of ALDH1A3 exhibited positive correlation with these tumor invasion associated genes (Fig 4D). Additionally, referring to its up-regulation in high-grade and Mes subtype gliomas, ALDH1A3 was supposed to be closely related to glioma cell invasion.

### ALDH1A3 silencing inhibits invasion of glioma cells in vitro

**Fig 5:**
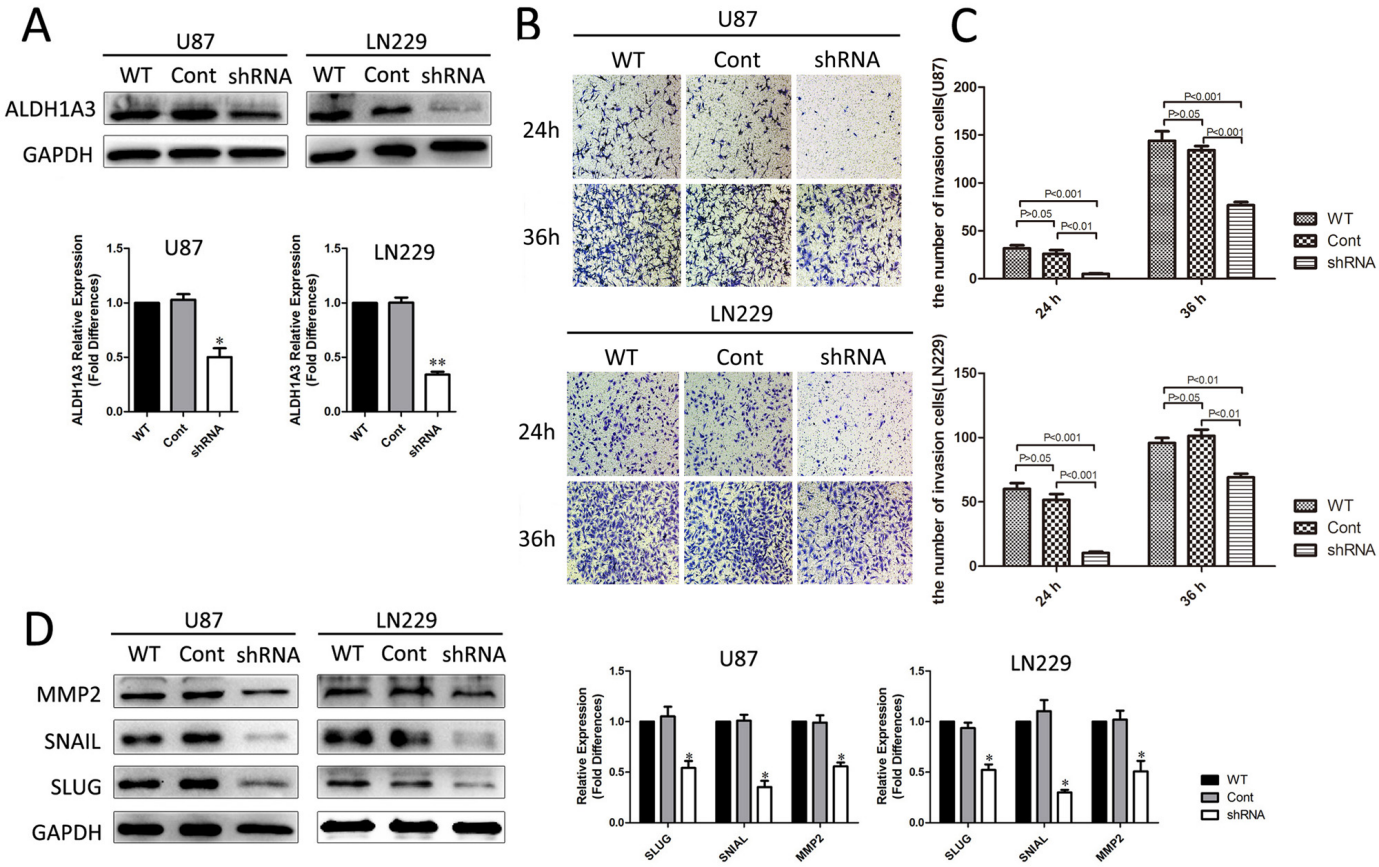
Knockdown of ALDH1A3 by shRNA inhibits invasion of glioma cells. **A**. After transduction, ALDH1A3 expression was determined by western blot. The expression of ALDH1A3 was significantly reduced by shRNA targeting ALDH1A3 as measured by densitometry (normalized to GAPDH). **B**. The number of invaded cells was significantly decreased in ALDH1A3 knockdown cells as compared with control. **C**. The quantitative results for the transwell experiment. **D**. The expression of tumor invasion associated proteins was reduced by ALDH1A3 knockdown. Each experiment was performed in triplicates. * p<0.05.

It is well known that malignant glioma cells are characteristic of high invasiveness. In our study, we assessed whether the invasiveness of glioma cells could be inhibited by ALDH1A3 silencing in vitro. By shRNA, ALDH1A3 was transiently knockdown in U87 and LN229 glioma cells. The decrease expression of ALDH1A3 was confirmed by western blot (Fig 5A). We used a transwell system to measure the ability of cells to invade the Matrigel and transmigrate across the membrane. As is shown in the figures (Fig 5B and 5C), after incubation for 24h or 36h, the ratio of invading cells decreased significantly in shRNA-pretreated U87 and LN229 cells. It indicated that the invasive abilities of the tumor cells were significantly suppressed by ALDH1A3 silencing. These results were also confirmed by ALDH1A3 knockdown in U87 and LN229 cells using shRNA, which resulted in the down-regulation of protein expression of invasion associated markers: SNAIL, SLUG and MMP2 (Fig 5D).

## Discussion

Gliomas are the most common group of primary malignant tumors of the central nervous system. Malignant gliomas have intrinsic resistance to current therapies that lead to extremely poor clinical outcomes. Thus, there is an urgent need to have better understanding of the underlying mechanisms of such malignancy, providing an opportunity to develop novel therapies and approaches to treat patients with aggressive gliomas.

ALDH is a group of enzymes that catalyzes the oxidation (dehydrogenation) of aldehydes. To date, 19 ALDH genes have been identified within the human genome. ALDH isoforms had been proved to be involved in a variety of biological process in various cancers and ALDH activity had been widely used as a universal CSCs marker [1, 23, 24]. But the functional ALDH isoforms and their clinical applications remained to be elucidated in gliomas. Thus, the investigation of ALDHs isoforms prevalence in gliomas and their related biological behaviors may be of great clinical applicability.

In our previously reported works, high ALDH1A3 mRNA expression had been proved to be related to poor clinical outcomes [10]. In the present study, we analyzed the ALDH1A3 mRNA expression in different grade gliomas demonstrated a significant increase from low- to high- grade gliomas. What’s more, patients with high ALDH1A3 mRNA expression had worse overall survival than those with low ALDH1A3 mRNA expression in HGG. These findings suggested that ALDH1A3 may be associated with the malignancy of gliomas and could serve as a prognostic biomarker.

ALDH1A1 was supposed to be another important ALDH isoform in many cancers. In gliomas, the effect of ALDH1A1 predicting the clinical outcome was controversial. Schafer A et al [5] reported that ALDH1A1 was a mediator for resistance of GBM to temozolomide and a reliable predictor of poor clinical outcome. But in another study, strong expression of ALDH1A1 was related to significantly better survival in GBM patients [6]. In our study, ALDH1A1 presented a significantly decreased expression in HGG than that in LGG gliomas. While, the prognostic capability of ALDH1A1 in HGG was poor. Thus, we think that ALDH1A1 may not be an ideal indicator in gliomas. However, its functions in gliomas still need to be further elucidated.

The preferential mRNA expression of ALDH1A3 in Mes subtype of gliomas is noteworthy. By transcriptome array analyses, Ping Mao et al [12] found that GSCs could be classified into two distinct subtypes, including PN GSCs and Mes GSCs. ALDH1A3 could be a potentially useful biomarker for Mes GSCs. In this study, we analyzed ALDH1A3 mRNA expression by large clinical samples from our CGGA data and further validated our findings in TCGA microarray database. Importantly, we found that ALDH1A3 was highly expressed in Mes subtypes, and could be a novel diagnostic marker for Mes gliomas. On the contrary, patients with ALDH1A3 mRNA low expression were more likely to be PN subtype. It was supposed that gliomas in specific subtypes could develop as the result of different cells of origin [25]. In addition to the fact that ALDH serving as a functional cancer stem cell marker in many human tumors, we speculate that gliomas with different ALDH1A3 mRNA expression level might have cells from different origins.

ALDH1A3 was reported to be highly expressed in many tumors with potential clinical prognostic applicability. But the biological functions of ALDH1A3 in gliomas were not well elucidated yet. In the current study, we found that the mRNA expression level of ALDH1A3 in gliomas was correlated to many Mes subtype markers and tumor invasion associated genes. Changes of cell adhesion were more likely related to ALDH1A3 mRNA expression. Knockdown of ALDH1A3 by shRNA constructs could markedly down-regulate these tumor invasion associated genes (SNAIL, SLUG and MMP2), and the abilitie of cell invasion was also reduced in vitro. In view of high expression in Mes gliomas and its function in cell invasion, ALDH1A3 may serve as a potential therapeutic target for Mes subtype gliomas.

However, further studies are still needed to elucidate the role of ALDHs in gliomas. Firstly, ALDH isoform(s) responsible for ALDH activity remains to be known. Secondly, an unresolved puzzle pertains to the precise role(s) of ALDH1A3 in tumor invasion. Thirdly, a recent study reported that inhibition of ALDH activity effectively eradicates drug-tolerant tumor cell subpopulations [26], which indicate a potential beneficial effect of combination therapy that includes ALDHs inhibition to delay gliomas relapse.

## Conclusion

We found that ALDH1A3 overexpression was significantly associated with high grade as well as the higher mortality of HGG. ALDH1A3 could also be a novel marker for Mes subtype gliomas. Moreover, the reduction of cell invasive ability in ALDH1A3 knockdown cell lines indicated that strategies designed to target ALDH1A3 might lead to more effective therapies for Mes subtype gliomas.

## Supporting Information

S1 Fig ALDH1A1 mRNA expression is decreased in high grade gliomas. By genome-wide transcriptome microarray (A) and whole transcriptome sequencing (B) analysis, the mRNA expression of ALDH1A1 is decreased in high grade gliomas. **C**. TCGA transcriptome sequencing data show that mRNA expression level of ALDH1A1 is lower in high grade gliomas than in low grade. Lines in the middle were the mean expression value.
(TIF)

S2 Fig Kaplan-Meier estimates of survival for patients (OS ≥ 60 days) with different ALDH1A1 expression. **A**. Kaplan-Meier estimates of survival for 177 HGG patients with genome-wide transcriptome microarray. There is no difference in OS between ALDH1A1 mRNA high-expression (n = 89, p>0.05) and low-expression patients (n = 88). **B**. In TCGA database, there is no difference in OS between ALDH1A1 mRNA high-expression (n = 220) and ALDH1A1 mRNA low-expression (n = 221, p>0.05) patients.
(TIF)
