# Supplementary figures and images for: ALDH1A3: A Marker of Mesenchymal Phenotype in Gliomas Associated with Cell Invasion
Source: PLoS One. 2015 Nov 17;10(11):e0142856. doi: 10.1371/journal.pone.0142856 (PMC4648511; doi:10.1371/journal.pone.0142856)

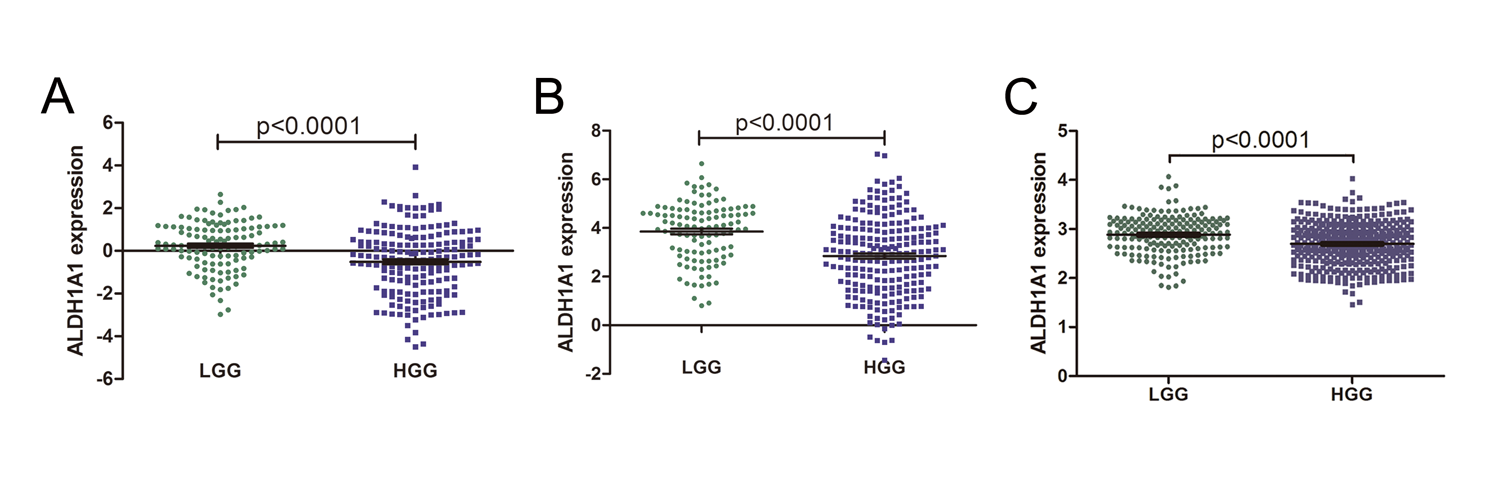

Supplement: S1 Fig — By genome-wide transcriptome microarray (A) and whole transcriptome sequencing (B) analysis, the mRNA expression of ALDH1A1 is decreased in high grade gliomas. C. TCGA transcriptome sequencing data show that mRNA expression level of ALDH1A1 is lower in high grade gliomas than in low grade. Lines in the middle were the mean expression value. (TIF) [file pone.0142856.s001.tif]

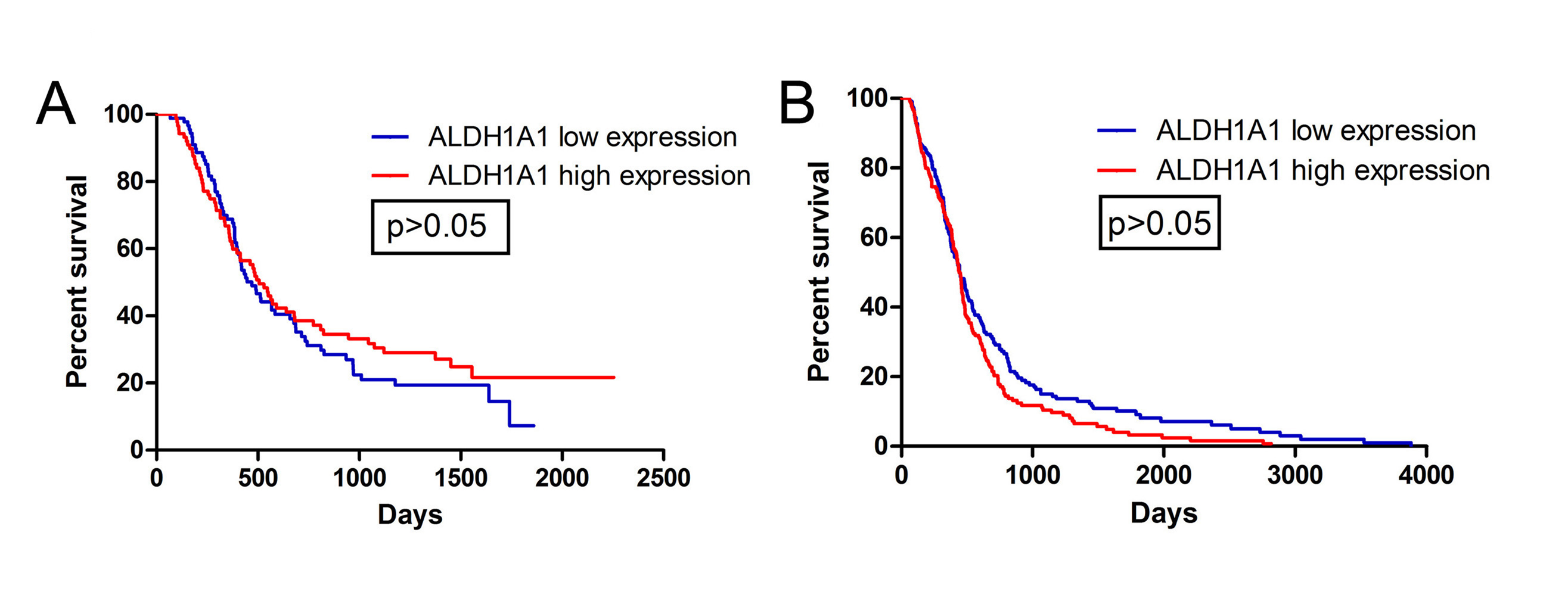

Supplement: S2 Fig — A. Kaplan-Meier estimates of survival for 177 HGG patients with genome-wide transcriptome microarray. There is no difference in OS between ALDH1A1 mRNA high-expression (n = 89, p>0.05) and low-expression patients (n = 88). B. In TCGA database, there is no difference in OS between ALDH1A1 mRNA high-expression (n = 220) and ALDH1A1 mRNA low-expression (n = 221, p>0.05) patients. (TIF) [file pone.0142856.s002.tif]
